# Effect of Random Base Vibrations on the Performance of Piezoelectric Wind Energy Harvesters

**DOI:** 10.3390/mi16121353

**Published:** 2025-11-28

**Authors:** Alberto Pasetto, Michele Tonan, Matteo Bottin, Alberto Doria

**Affiliations:** Department of Industrial Engineering, University of Padova, 35131 Padova, Italy; alberto.pasetto.2@phd.unipd.it (A.P.); michele.tonan@unipd.it (M.T.); matteo.bottin@unipd.it (M.B.)

**Keywords:** piezoelectric vibration harvester, vortex-induced vibration (VIV), galloping, random base motion

## Abstract

Piezoelectric wind energy harvesters can collect a small amount of energy from wind without the need for rotary equipment. In practice, such harvesters can be excited concurrently by wind-induced and base vibrations. In this study, combined wind and base excitation is investigated, with a focus on random base vibrations and the effect of the bandwidth of band-limited random excitation, thereby filling the research gap between results obtained with wide-band random excitation and those with harmonic excitation. Since flow-induced vibrations can produce several phenomena, in this research, galloping and vortex-induced vibration (VIV) harvesters are considered due to their structural similarity and the ease with which a galloping harvester can be converted into a VIV harvester (and vice versa). Both numerical and experimental results are presented. First, the mathematical models are given; then, experimental tests validate the models and provide an insight into the phenomena; finally, numerical simulations extend the dissertation by providing a more in-depth analysis of the behavior of such harvesters. The results show that above the critical wind velocity, galloping harvesters are not affected by the amplitude and bandwidth of random base excitation. In contrast, VIV harvesters in the lock-in condition are affected by random base excitation, especially if the vibration amplitude is large and if its spectrum is concentrated in a narrow band centered about the resonance.

## 1. Introduction

Wind energy has been exploited for many centuries; it is documented that windmills were used in Europe and Persia during the Middle Ages. In recent years, decarbonization efforts have led to significant improvements in wind turbines, and today, over 10% of energy is produced by wind farms [1].

The development of new technologies has accelerated not only the development of wind turbines for the massive production of energy, but also the ideation of small devices that collect small amounts of energy from wind without using rotary equipment, called wind harvesters. These devices are useful for feeding sensor nodes and small electronic components and will play an important role in the internet of things (IoT).

Wind harvesters first convert the kinetic energy of wind into the kinetic energy of vibration of an elastically suspended body. Then, they convert the vibration energy into useful electrical energy, exploiting physical phenomena such as electromagnetism, piezoelectricity, and triboelectricity [2].

Piezoelectric conversion is based on piezoelectric layers that can be easily integrated into a vibrating structure, are cost-effective, and have a high electro-mechanical coupling factor. Hence, it appears to be the most promising mechanical–electrical conversion technology for small and micro devices [3,4] and will be considered throughout this paper.

In particular, in this study, a patch made of PZT (lead zirconate titanate) will be analyzed, as it has good piezoelectric properties and is a low-cost device [5].

Wind energy can be converted into mechanical energy without making use of rotary machines by exploiting flow-induced vibrations [6]. In recent years, research has aimed to exploit several phenomena, such as galloping [7,8], vortex-induced vibrations (VIVs) [9,10], flutter [11,12], and turbulence [13], to develop wind-excitation harvesters. Some prototypes capable of scavenging significant amounts of energy have been developed and tested [14].

There are many actual implementations of wind harvesters in which the piezoelectric harvesters are concurrently excited by both wind-induced vibrations and base vibrations. This condition is rather common for the harvesters mounted on railways [15], bridges [16], buoys [17], and light vehicles [18]. In wind harvesters, since the conversion of fluid energy into mechanical vibration energy is based on non-linear phenomena, when both base and wind excitation are present, the superposition principle does not hold true, and there is a possibility that base vibrations will have a negative effect on the aero-elastic phenomena, with a possible reduction in the generated voltage.

Some researchers have developed hybrid harvesters that are suited to concurrent base and wind excitation. Hybrid galloping harvesters composed of a piezoelectric cantilever beam clamped at one end and fixed to a square-sectioned bluff body at the other end have been studied both numerically and experimentally in [19]. Results obtained using harmonic base excitation showed that above the velocity of galloping instability, base excitation has a beneficial effect on the generated voltage only when its frequency is very close to the resonance frequency. If the base excitation frequency is farther from the resonance frequency, the response from the combined loading (base and wind excitation) can sometimes drop below that from the aerodynamic load alone, and the time-domain signals exhibit quasi-periodic oscillations. Finally, when the base excitation frequency is far from resonance, it has a negligible effect on the combined response. In [20], the analysis of the effect of combined harmonic base excitation and wind excitation was extended to a cantilever harvester equipped with a cylinder bluff body with two attachments. More complex galloping harvesters under concurrent wind and harmonic base excitation were studied in [21,22]. Recently, in [23], the transition between periodicity, quasi-periodicity, and chaos was studied in a hybrid galloping harvester equipped with a D-shaped bluff body. The presence of harmonic base excitation and random wind fluctuations was considered.

VIV harvesters are similar to galloping harvesters, but the bluff body is a cylinder. The first study on the effect of concurrent vortex-induced vibration and harmonic base excitation was performed in [24]. Numerical results showed that, when the vortex shedding frequency and the resonance frequency coincide (known as the lock-in condition), the level of generated power is larger than that generated by the two excitations separately. In [25], the analysis was extended to two-degrees-of-freedom VIV harvesters with harmonic base excitation; the numerical results showed that the presence of base excitation increased the bandwidth and the generated voltage incrementally.

An experimental analysis of the effect of concurrent harmonic base excitation and flutter is presented in [26], showing that flutter vibrations make a small contribution to the large voltage generated by base excitation.

It is important to highlight that the above-mentioned research has analyzed the effect of harmonic base excitation on the performance of several kinds of harvesters. However, up to now, the effect of random base vibrations has been scarcely addressed. In [17], galloping harvesters mounted on a buoy platform, excited both by aerodynamic forces and random waves, were studied. The numerical and analytical results showed that when the wind velocity exceeds the galloping critical velocity, the response is large, periodic, and dominated by galloping oscillations. At lower wind velocity, the contribution of random base excitation is more important. In [27], the dynamics of a galloping harvester with an RLC circuit under random excitation were studied. The numerical and analytical results showed that the generated power increases with the amplitude of random base excitation, and the effect is more pronounced at low wind velocities. The effect of random base vibrations on VIV harvesters has scarcely been studied. Some results obtained using broad-band white noise are presented in [28]. In [18], the effect of random road-induced vibrations on the performance of a VIV harvester mounted on a bicycle was studied. The simulated results showed that, even though the system is non-linear, under lock-in conditions, the effects of aerodynamic excitation and random road excitation almost add up.

This paper aims to fill the research gap in the field of wind harvesters excited by random base vibrations, considering the effect of the bandwidth of band-limited random excitation. This study is interesting not only for its practical applications, but also because reducing the bandwidth of random excitation enables filling the gap between results obtained with wide-band random excitation and those with harmonic excitation. Moreover, given the few experimental results on combined wind and base excitation in the literature, some experimental tests are presented to validate the models. In this research, galloping and VIV harvesters are considered because they are very similar from a constructive point of view, and a VIV harvester can be converted into a galloping harvester (and vice versa) simply by modifying the shape of the bluff body, without modifying the cantilever or the piezoelectric layer.

The rest of the paper is organized as follows. In Section 2, the layout of galloping and VIV harvesters is described. In Section 3, the mathematical model is presented. The mechanical and piezoelectric models of the galloping and VIV harvesters are identical. In contrast, two different expressions of the aerodynamic force make it possible to simulate the two different aerodynamic sources of excitation. Section 4 provides the experimental results obtained in the presence of concurrent wind and base excitation. Wind excitation was generated by means of a wind tunnel, whereas random base excitation was generated by means of an electromagnetic shaker. Section 5 deals with the numerical simulations of galloping harvesters, and Section 6 deals with the numerical simulation of VIV harvesters. In both cases, the effect of random excitation bandwidth and amplitude is analyzed and discussed. Finally, conclusions are drawn in Section 7.

## 2. Harvester Layouts

**Figure 1:**
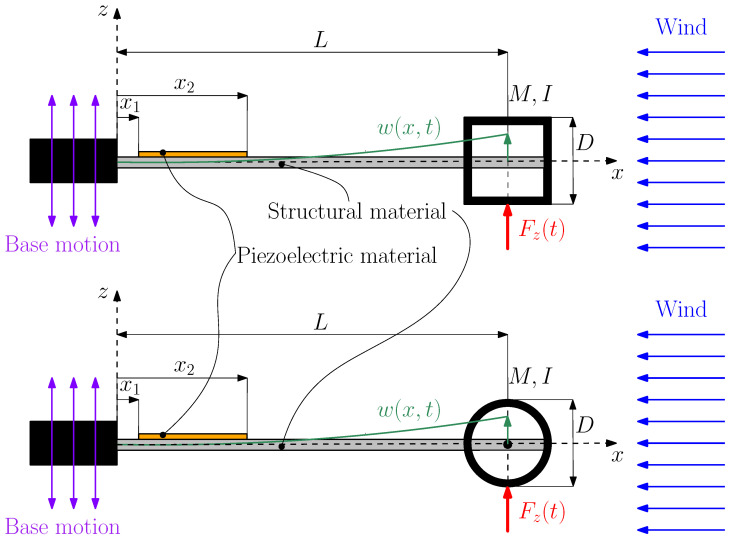
Scheme of the piezoelectric harvester equipped with a square-section parallelepiped bluff body for galloping excitation (**top**), as well as with a cylindrical bluff body for VIV excitation (**bottom**).

Figure 1 shows the scheme of the galloping and VIV harvesters. The harvesters are composed of three different components. The first element is the structural beam, which provides mechanical support and defines the system’s dynamic behavior. *L* represents the length of the structural material from the clamp to the center of the bluff body. The second component is the piezoelectric patch, bonded to the beam surface, which converts mechanical strain into electrical energy through the direct piezoelectric effect. The patch is bounded at a distance *x*_1_ from the clamp, ending at *x*_2_. The third component is the bluff body, which has two key functions: first, it lowers the beam’s natural frequency by increasing its effective mass, and second, it generates aerodynamic force. In the case of galloping, the square-sectioned bluff body induces aerodynamic instability, whereas in the case of VIV, the cylindrical bluff body makes vortex shedding possible. *D* represents the side of the square-section parallelepiped bluff body and the diameter of the cylindrical bluff body. The bluff bodies have the same mass; hence, both harvesters have the same natural frequency.

## 3. Mathematical Model

### 3.1. Electro-Mechanical Model of the Cantilever

Aerodynamic forces and base acceleration excite the vibrations of the cantilever beam and are described by the following partial derivative equation:

(1) $$\begin{array}{cc} \; & EJ\frac{\partial^{4}w\left(x,t\right)}{\partial x^{4}}+E^{*}J\frac{\partial^{5}w\left(x,t\right)}{\partial x^{4}\partial t}+m\frac{\partial^{2}w\left(x,t\right)}{\partial t^{2}}+c_{a}\frac{\partial w(x,t)}{\partial t}+\theta \;v\left(t\right)\;\left[\frac{d\delta (x-x_{1})}{dx}-\frac{d\delta (x-x_{2})}{dx}\right]=\\ \; & F_{z}\left(t\right)\delta \left(x-L\right)-M\frac{d^{2}w_{B}\left(t\right)}{dt^{2}}\delta \left(x-L\right)-m\frac{d^{2}w_{B}\left(t\right)}{dt^{2}}-c_{f}\frac{dw_{T}\left(t\right)}{dt}\delta \left(x-L\right) \end{array}$$

*t* is the time, coordinate *x* represents the position along the beam, w(x,t) is the transverse displacement of any point of the cantilever with respect to the clamp, and v(t) is the voltage. *m* is the mass per unit length of the beam, EJ is the bending stiffness of the cross-section, *J* is the equivalent area moment of inertia of the cross-section, $E^{*}$ is a time-independent visco-elastic parameter [29], and $c_{a}$ is the viscous damping coefficient of the beam. The two damping terms are assumed to satisfy the proportional damping criteria [30]. δ is Dirac’s delta. Owing to the piezoelectric effect, Equation (1) depends on voltage through the electro-mechanical coupling coefficient (θ), defined by the following equation:

(2) $$\theta =\frac{e_{31}\;b}{2\;h_{p}}\;\left(h_{b}^{2}-h_{c}^{2}\right)$$

where $e_{31}$ is the piezoelectric constant, $h_{p}$ is the thickness of the piezoelectric patch, $h_{b}$ is the position of the bottom of the piezoelectric patch from the neutral axis of the composite cross-section, $h_{c}$ is the position of the top of the piezoelectric patch from the neutral axis, and *b* is the width of piezoelectric patch. The presence of the clamp and the bluff body is taken into account by setting proper boundary conditions at the two ends of the beam [31].

The right-hand side of Equation (1) contains the following forcing terms:

- $F_{z}\left(t\right)\delta \left(x-L\right)$ is the lumped aerodynamic force due to galloping or to vortex shedding; it is applied to the center of the cylinder.
$-M\frac{d^{2}w_{B}\left(t\right)}{dt^{2}}\delta \left(x-L\right)$ is the lumped force of inertia due to the bluff body mass.
$-m\frac{d^{2}w_{B}\left(t\right)}{dt^{2}}$ is the distributed force of inertia due to the cantilever mass.
$-c_{f}\frac{dw_{T}\left(t\right)}{dt}\delta \left(x-L\right)$ is the lumped force due to the fluid-added damping of the bluff body [6,32], which depends on the absolute velocity of the bluff body $\left(\frac{dw_{T}\left(t\right)}{dt}\right)$. This velocity is the sum of the base velocity and the relative velocity of the bluff body due to cantilever bending: $\frac{dw_{T}\left(t\right)}{dt}=\left(\frac{dw_{B}\left(t\right)}{dt}+\frac{\partial w(x,t)}{\partial t}{|}_{x=L}\right)$. Coefficient $c_{f}$ represents the fluid-added damping of the cylinder and is given by [32]
  (3) $$c_{f}=2\pi \gamma \mathrm{St}\rho UDL_{T}$$
  where ρ is the fluid density, γ is a non-dimensional parameter [6], $L_{T}$ is the length of the bluff body, and St is the Strouhal number. It is worth noting that this term is introduced in the VIV models [32], but is not included in the galloping models.

The modal expansion approach makes it possible to represent the transverse displacement w(x,t) of the cantilever harvester as a series of eigenfunctions:

(4) $$w\left(x,t\right)=\sum_{m=1}^{\infty }\Psi_{m}\left(x\right)\;\eta_{m}\left(t\right)$$

where $\Psi_{m}\left(x\right)$ is the *m*th mode of vibration and $\eta_{m}\left(t\right)$ is the *m*th modal displacement. The modes of vibration are obtained by solving the eigenvalue problem related to Equation (1) and by considering the boundary conditions due to the presence of the clamp at x=0 and the bluff body at x=L. The modes of vibration are then normalized by setting the tip amplitude to one ($\Psi_{m}\left(L\right)=1$).

By inserting Equation (4) into Equation (1) and exploiting the orthogonality conditions, the modal equations of the cantilever harvester are obtained:

(5) $$\begin{array}{cc} \; & m_{n}\;\frac{d^{2}\eta_{n}\left(t\right)}{dt^{2}}+c_{n}\;\frac{d\eta_{n}\left(t\right)}{dt}+k_{n}\eta_{n}\left(t\right)+\varphi_{n}v\left(t\right)=\\ \; & \int_{0}^{L}F_{z}\left(t\right)\delta \left(x-L\right)\Psi_{n}\left(x\right)dx-\int_{0}^{L}M\frac{d^{2}w_{B}\left(t\right)}{dt^{2}}\delta \left(x-L\right)\Psi_{n}\left(x\right)dx-\int_{0}^{L}m\frac{d^{2}w_{B}\left(t\right)}{dt^{2}}\Psi_{n}\left(x\right)\;dx+\\ \; & -\int_{0}^{L}c_{f}\frac{dw_{B}\left(t\right)}{dt}\delta \left(x-L\right)\Psi_{n}\left(x\right)dx-\int_{0}^{L}c_{f}\sum_{m=1}^{\infty }\Psi_{m}\left(L\right)\;{\dot{\eta }}_{m}\delta \left(x-L\right)\Psi_{n}\left(x\right)dx\\ \; & \;\mathrm{with}\;n=1,\ldots ,\infty \end{array}$$

After calculating the integrals, the modal equations become

(6) $$\begin{array}{cc} \; & m_{n}\;{\ddot{\eta }}_{n}+c_{n}\;{\dot{\eta }}_{n}+k_{n}\eta_{n}+\varphi_{n}v=\\ \; & F_{z}\Psi_{n}\left(L\right)-M{\ddot{w}}_{B}\Psi_{n}\left(L\right)-m{\ddot{w}}_{B}\int_{0}^{L}\Psi_{n}\left(x\right)\;dx-c_{f}{\dot{w}}_{B}\Psi_{n}\left(L\right)-c_{f}\Psi_{n}\left(L\right)\sum_{m=1}^{\infty }\Psi_{m}\left(L\right)\;{\dot{\eta }}_{m}\\ \; & \;\mathrm{with}\;n=1,\ldots ,\infty \end{array}$$

where the symbols $\dot{\left(.\right)}$ and $\ddot{\left(.\right)}$ indicate the first and second time derivatives. $m_{n}$, $c_{n}$, and $k_{n}$ are the modal mass, damping, and stiffness of the *n*th mode of vibration, which are given by the following equations:

(7) $$\begin{array}{cc} m_{n}= & \int_{0}^{L}\Psi_{n}\left(x\right)\;m\Psi_{n}\left(x\right)\;dx+M\Psi_{n}{\left(L\right)}^{2}+\frac{d\Psi_{n}\left(x\right)}{dx}{|}_{x=L}I\frac{d\Psi_{n}\left(x\right)}{dx}{|}_{x=L} \end{array}$$

(8) $$k_{n}=\int_{0}^{L}\frac{d^{2}\Psi_{n}\left(x\right)}{dx^{2}}EJ\frac{d^{2}\Psi_{n}\left(x\right)}{dx^{2}}dx$$

(9) $$c_{n}=2\zeta_{n}\sqrt{k_{n}m_{n}}$$

Constant $\zeta_{n}$ in Equation (9) is the modal damping ratio of the *n*th mode of vibration. In Equations (5) and (6), $\varphi_{n}$ is the backward piezoelectric modal coupling term (in the selected harvester configuration, it is equal to the forward modal coupling term), which is given by [33]

(10) $$\varphi_{n}=\theta \;\left(\frac{d\Psi_{n}\left(x\right)}{dx}{|}_{x=x_{2}}-\frac{d\Psi_{n}\left(x\right)}{dx}{|}_{x=x_{1}}\right)$$

The voltage generated by the piezoelectric cantilever can be calculated as described in [33]

(11) $$C_{p}\dot{v}+\frac{v}{R}=\sum_{m=1}^{\infty }\varphi_{m}{\dot{\eta }}_{m}$$

where $C_{p}$ is the capacitance of the piezoelectric layer and *R* is the load resistance. In the open circuit condition (R→∞), Equation (11) becomes

(12) $$v=v_{0}+\frac{1}{C_{p}}\sum_{m=1}^{\infty }\varphi_{m}\eta_{m}$$

where $v_{0}$ is the open circuit voltage (OCV) in the initial condition.

Finally, if Equation (12) is inserted into Equation (6) with $v_{0}=0$, the electromechanical modal equations become

(13) $$\begin{array}{cc} \; & m_{n}\;{\ddot{\eta }}_{n}+c_{n}\;{\dot{\eta }}_{n}+c_{f}\Psi_{n}\left(L\right)\sum_{m=1}^{\infty }\Psi_{m}\left(L\right)\;{\dot{\eta }}_{m}+k_{n}\eta_{n}+\varphi_{n}\frac{1}{C_{p}}\sum_{m=1}^{\infty }\varphi_{m}\eta_{m}=\\ \; & F_{z}\Psi_{n}\left(L\right)-M{\ddot{w}}_{B}\Psi_{n}\left(L\right)-m{\ddot{w}}_{B}\int_{0}^{L}\Psi_{n}\left(x\right)\;dx-c_{f}{\dot{w}}_{B}\Psi_{n}\left(L\right)\\ \; & \;\mathrm{with}\;n=1,\ldots ,\infty \end{array}$$

In most cantilever energy harvesters, the generation of electric energy is chiefly due to the excitation of the first bending mode of vibration [17,19,20,27]; the higher-order modes have negligible effects [34]. Therefore, only the first mode of vibration is retained in the model, and the equation of motion becomes

(14) $$\begin{array}{cc} \; & m_{1}\;{\ddot{\eta }}_{1}+c_{1}\;{\dot{\eta }}_{1}+c_{f}\Psi_{1}^{2}\left(L\right)\;{\dot{\eta }}_{1}+k_{1}\eta_{1}+\frac{\varphi_{1}^{2}}{C_{p}}\eta_{1}=\\ \; & F_{z}\Psi_{1}\left(L\right)-M{\ddot{w}}_{B}\Psi_{1}\left(L\right)-m{\ddot{w}}_{B}\int_{0}^{L}\Psi_{1}\left(x\right)\;dx-c_{f}{\dot{w}}_{B}\Psi_{1}\left(L\right) \end{array}$$

### 3.2. Force Due to Galloping

**Figure 2:**
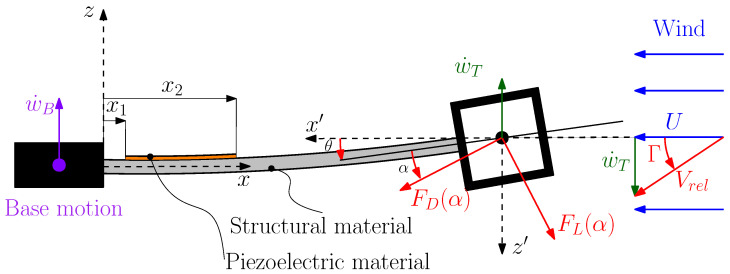
Angle of attack and aerodynamic forces.

The galloping phenomenon occurs because the vibrations of the bluff body cause a variation in the angle of attack between the bluff body and the incoming wind (see Figure 2), this results in a variation in the lift and drag forces, which is defined by the following equations:

(15) $$F_{D}\left(t\right)=\frac{1}{2}\;\rho DL_{T}V_{rel}^{2}C_{D}\left(\alpha \right)$$

(16) $$F_{L}\left(t\right)=\frac{1}{2}\;\rho DL_{T}V_{rel}^{2}C_{L}\left(\alpha \right)$$

In Equations (15) and (16) $V_{rel}$, the relative wind velocity is $C_{D}$, and $C_{L}$ represents the drag and lift coefficients, which depend on the angle of attack α.

The bluff body is mounted at the free end of the cantilever; therefore, the variation in the angle of attack takes place owing to two phenomena: the variation in the end slope of the cantilever due to bending, and the variation in the relative velocity direction caused by the translational velocity of the bluff body [19].

In order to define the angle of attack, a reference frame $x^{\prime }y^{\prime }z^{\prime }$ is introduced. Its origin is at the center of the bluff body, and axis $x^{\prime }$ is always aligned to wind velocity *U*. When the cantilever bends, the chord of the bluff body makes an angle θ with $x^{\prime }$, which is given by

(17) $$\theta =\frac{\partial \;w(x,t)}{\partial x}{|}_{x=L}$$

Figure 2 shows that when the bluff body moves upwards with velocity ${\dot{w}}_{T}\left(t\right)$, the relative velocity of the incoming wind $V_{rel}$ is tilted by angle Γ with respect to the absolute wind velocity *U*. If small amplitude vibrations are considered, angle Γ in coordinate system $x^{\prime }y^{\prime }z^{\prime }$ is given by the following equation:

(18) $$\Gamma =\frac{{\dot{w}}_{T}\left(t\right)}{U}=\frac{\left({\dot{w}}_{B}\left(t\right)+\frac{\partial w(x,t)}{\partial t}{|}_{x=L}\right)}{U}$$

Therefore, the angle of attack, which is the angle between the wind relative velocity and the chord, is

(19) α=Γ−θ

If the expressions of Γ and θ are introduced into (19), the angle of attack becomes

(20) $$\alpha =\frac{\left({\dot{w}}_{B}\left(t\right)+\frac{\partial w(x,t)}{\partial t}{|}_{x=L}\right)}{U}-\frac{\partial \;w(x,t)}{\partial x}{|}_{x=L}$$

Angle α makes the calculation of the lift and drag forces, which are aligned with and perpendicular to the relative velocity, respectively, possible. The projection of the lift and drag forces along the *z* axis of frame xyz gives the aerodynamic force $F_{z}$ that appears in Equation (1). This force is usually expressed [19] in terms of a lateral force coefficient $C_{Fz}\left(\alpha \right)$:

(21) $$F_{z}\left(t\right)=\frac{1}{2}\;\rho DL_{T}U^{2}C_{Fz}\left(\alpha \right)$$

The coefficient $C_{Fz}\left(\alpha \right)$ depends on $C_{D}\left(\alpha \right)$ and $C_{L}\left(\alpha \right)$. In most studies dealing with galloping vibrations [7,8,23], an approximate polynomial formula of $C_{Fz}\left(\alpha \right)$ is adopted:

(22) $$C_{Fz}\left(\alpha \right)=a_{1}\alpha +a_{3}\alpha^{3}$$

Empirical coefficients $a_{1}$ and $a_{3}$ can be found in the scientific literature and are derived from the fitting of experimental results.

Finally, if the velocities and the slope in the expression of angle α are calculated by adopting the modal expansion of Equation (4) truncated at the first term, the following equation of the aerodynamic galloping force is obtained:

(23) $$\begin{array}{c} F_{z}\left(t\right)=\frac{1}{2}\;\rho DL_{T}U^{2}\left\{a_{1}\left(\frac{{\dot{w}}_{B}\left(t\right)+\Psi_{1}\left(L\right)\;{\dot{\eta }}_{1}}{U}-{\left.\frac{d\Psi_{1}\left(x\right)}{dx}\right|}_{x=L}\eta_{1}\right)+\right.\\ \left.+a_{3}{\left(\frac{{\dot{w}}_{B}\left(t\right)+\Psi_{1}\left(L\right)\;{\dot{\eta }}_{1}}{U}-{\left.\frac{d\Psi_{1}\left(x\right)}{dx}\right|}_{x=L}\eta_{1}\right)}^{3}\right\} \end{array}$$

The galloping force includes a linear part and a non-linear part. The linear part depends on $\eta_{1}$, ${\dot{\eta }}_{1}$, and ${\dot{w}}_{B}$. When the linear terms in $\eta_{1}$, ${\dot{\eta }}_{1}$ are moved to the left side of Equation (14), due to their signs, they generate a decrease in the linear damping coefficient of the system ($c_{lin}$) and an increase in the linear stiffness of the system ($k_{lin}$), which becomes

(24) $$c_{lin}=c_{1}-a_{1}\frac{1}{2}\rho DL_{T}U\Psi_{1}^{2}\left(L\right)$$

(25) $$k_{lin}=k_{1}+\frac{\varphi_{1}^{2}}{C_{p}}+a_{1}\frac{1}{2}\rho DL_{T}U^{2}\frac{d\Psi_{1}\left(x\right)}{dx}{|}_{x=L}$$

The non-linear part generates cubic terms in ${\dot{\eta }}_{1}$ and ${\dot{w}}_{B}$, a cubic term in $\eta_{1}$, and mixed terms. When the cubic terms in ${\dot{\eta }}_{1}$ and $\eta_{1}$ are moved to the left side of Equation (14), they generate cubic damping and stiffness terms, respectively. In contrast, the mixed terms generate a complex coupling between base motion and cantilever deflection. The cubic damping term becomes important at high tip velocities, and since $a_{3}$ is negative, it limits the vibration amplitude above the critical wind velocity.

### 3.3. Force Due to Vortex Shedding

A cylindrical bluff body is axisymmetric; hence, there are no variations in the lift and drag coefficients when the orientation of the bluff body changes with respect to the relative wind velocity. However, an alternate vortex shedding phenomenon occurs. The fluid motion around the cylinder and the vibrations are coupled because vortex shedding generates a fluctuating force ($F_{z}$), and in turn, cylinder vibrations affect vortex shedding. The force acting on the cylinder is given by

(26) $$F_{z}\left(t\right)=\frac{1}{2}\;\rho DL_{T}U^{2}C_{V}\left(t\right)$$

where $C_{V}\left(t\right)$ is a time-dependent lift coefficient related to vortex shedding. The fluid is a continuous system; hence, the effect of cylinder vibrations on vortex shedding is complex. To simplify the mathematical model of vortex shedding, reduced-order models are widely adopted in the scientific literature. In this study, the influence of cylinder vibrations on coefficient $C_{V}\left(t\right)$ is modeled by a non-linear van der Pol oscillator excited by the acceleration of the cylinder, following the approach of Facchinetti et al. [32]:

(27) $${\ddot{C}}_{V}+\epsilon \omega_{S}\left({\left(\frac{2C_{V}}{C_{V0}}\right)}^{2}-1\right){\dot{C}}_{V}+\omega_{S}^{2}C_{V}=\frac{AC_{V0}}{2D}{\ddot{w}}_{T}$$

In Equation (27), $C_{V0}$ is the lift coefficient related to the vortex shedding of the non-vibrating cylinder, $\omega_{S}=2\pi \mathrm{St}\frac{U}{D}$ is the angular frequency of vortex shedding, and ϵ and *A* are constants. The term on the right-hand side of the equation is the acceleration of the cylinder due to beam bending and base vibrations. If the acceleration of the cylinder due to beam bending $\left(\frac{\partial^{2}w\left(x,t\right)}{\partial t^{2}}{|}_{x=L}\right)$ is expressed by means of the truncated modal expansion (Equation (4)), the following equation holds:

(28) $${\ddot{C}}_{V}+\epsilon \omega_{S}{\left(\frac{2C_{V}}{C_{V0}}\right)}^{2}{\dot{C}}_{V}-\epsilon \omega_{S}{\dot{C}}_{V}+\omega_{S}^{2}C_{V}=\frac{AC_{V0}}{2D}\left(\Psi_{1}\left(L\right)\;{\ddot{\eta }}_{1}+{\ddot{w}}_{B}\right)$$

It is worth noting that Equation (28) includes a linear and a non-linear term in ${\dot{C}}_{V}$. The linear term is negative and generates self-sustained oscillations, whereas the non-linear term is positive, represents energy dissipation, and becomes important for large values of $C_{V}$. A limit cycle is reached when a balance between the linear and non-linear terms is reached.

## 4. Experimental Validation of the Numerical Model

**Figure 3:**
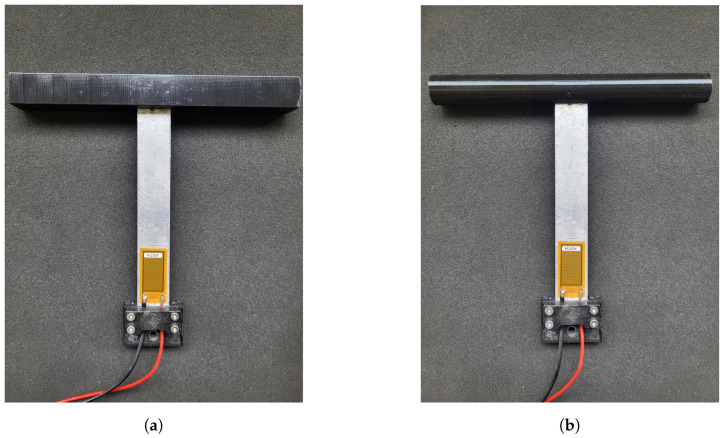
Experimental setup: (**a**) cantilever harvester equipped with a square bluff body (galloping), and (**b**) cantilever harvester equipped with a cylindrical bluff body (VIV).

To validate the mathematical model of the harvester subjected to galloping or VIV, some experimental tests were conducted. In the framework of this research, the piezoelectric energy harvesters represented in Figure 3a,b were used. The structural substrate is an aluminum cantilever beam (length: 140 mm; section: 21 × 1 mm). The piezoelectric active patch is made of Macro Fiber Composite and is manufactured by Smart Material GmbH (Dresden, Germany); it is 28 mm long and 14 mm wide. The patch is glued near the clamp where the maximum strain occurs.

As described in Section 2, the piezoelectric cantilever was equipped with a square-section parallelepiped bluff body (Figure 3a) for the galloping excitation and a cylindrical bluff body (Figure 3b) for the VIV excitation. The two bluff bodies have the same mass of 7.5 grams and are 178 mm long. The diameter of the cylindrical bluff body and the side of the square-section parallelepiped are 19 mm. The projected area of the two bluff bodies is the same.

The tests were carried out in a small wind tunnel in order to obtain a steady airflow. The test section of the wind tunnel measures 19 × 20 mm and has a total length of 80 cm. The maximum wind velocity is 4 m/s.

Considering a bluff body length of 178 mm, the blockage ratio in the wind tunnel experiments is approximately 9%, which indicates that blockage effects have a negligible influence on the galloping and VIV phenomena [35]. Moreover, another key parameter is the clearance between the cylinder ends and the wind tunnel walls, which corresponds to 58% of the dimension *D* = 19 mm. This spacing is sufficiently large to minimize wall interference, even accounting for boundary layer formation along the tunnel walls [36,37].

The voltage signal was recorded via a National Instruments acquisition board (NI 9230) (National Instruments Corp., Austin, Texas, US). Since the acquisition board’s input impedance is limited, an additional resistor was connected in series with the board to approximate open circuit conditions. Data recording was carried out at a sampling rate of 2048 Hz over a duration of 10 seconds. Each experimental test condition was replicated three times.

**Figure 4:**
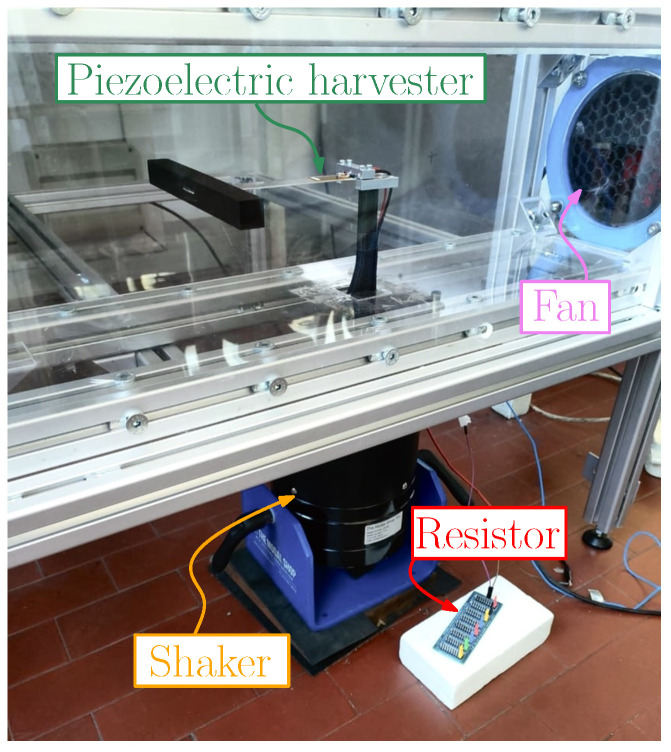
Cantilever harvester equipped with a square parallelepiped bluff body mounted on the shaker inside the wind tunnel.

The random base excitation was exerted by a Modal Shop 2075E Electrodynamic Shaker (The Modal Shop Inc., Cincinnati, OH, USA) driven by a Smartamp Power Amplifier (Series 2100E21) (The Modal Shop Inc., Cincinnati, OH, USA), as depicted in Figure 4.

**Figure 5:**
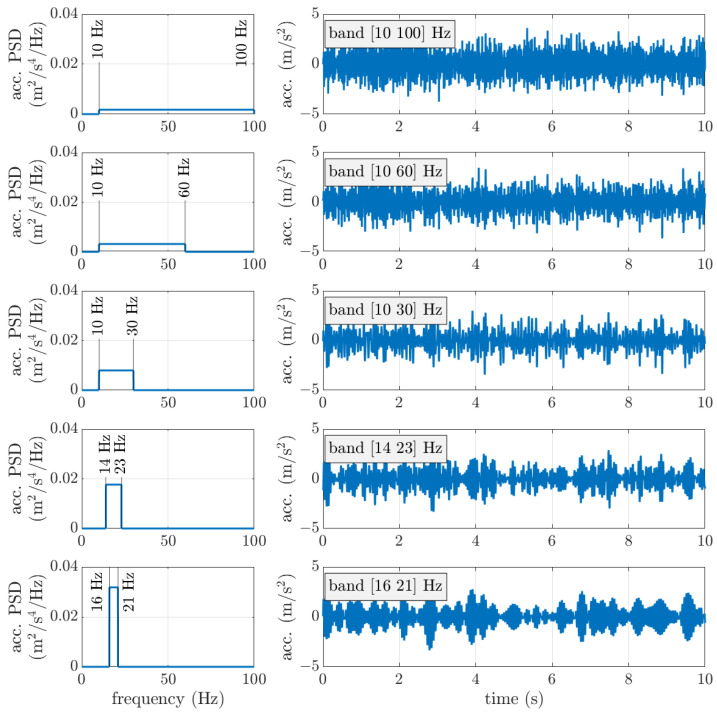
PSDs of band-limited white noise, and examples of the corresponding base acceleration signals (rms equal to 1 m/s^2^).

Base excitation was assumed to be band-limited white noise with assigned rms value and constant PSD (power spectral density). In order to generate band-limited white noise in the time domain, the spectral representation method was adopted [38]. Base acceleration consists of *N* harmonics in the frequency band [*f_min_* *f_max_*], with random phases and amplitudes $a_{n}$ given by

(29) $$a_{n}=\sqrt{\mathrm{PSD}(n\;\Delta f)\;\Delta f}$$

where $\Delta f=\frac{f_{max}-f_{min}}{N-1}$. In particular, N=2400 harmonics were considered in the band [0 100 Hz]. The acceleration PSDs used to generate the signals are depicted in Figure 5 together with examples of random acceleration signals.

### 4.1. Experimental Validation of the Galloping

**Figure 6:**
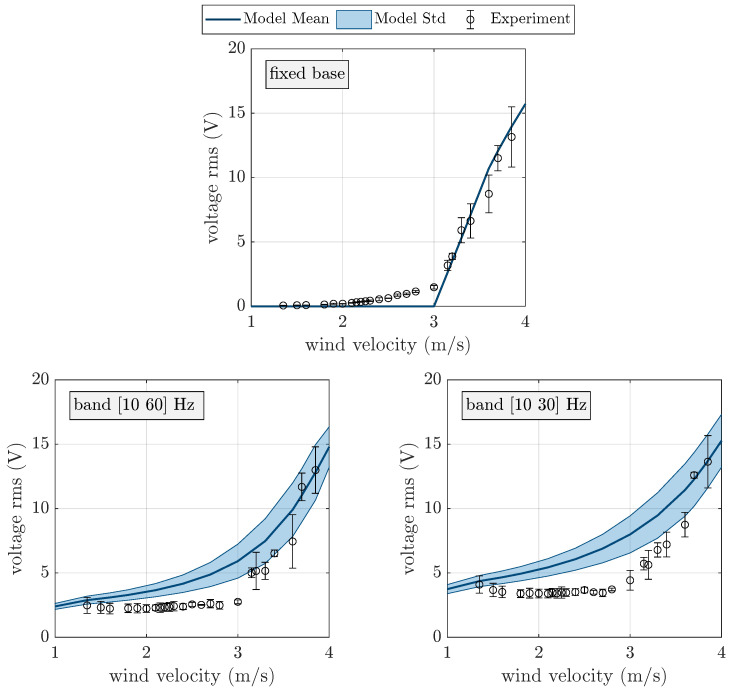
Experimental and numerical OC voltage of the galloping harvester without base excitation and with band-limited random base excitation (rms 1 m/s^2^).

The results of the experimental tests carried out with the galloping harvester are depicted in Figure 6 in terms of open circuit (OC) voltage rms versus wind velocity. Three different working conditions are considered: only wind excitation; wind excitation plus random base excitation in the band [10 60] Hz and an rms value of 1 m/s^2^; wind excitation plus random base excitation in the band [10 30] Hz and an rms value of 1 m/s^2^. Since every experimental test was repeated three times, for every value of wind velocity, the mean value and the standard deviation of the rms voltage are represented.

Figure 6 shows that, when only wind excitation is present, the rms amplitude of the OC voltage is negligible up to 2 m/s. Then, there is a modest increase in voltage amplitude, which can be caused by a reduction in total damping due to aerodynamic effects [19]. Finally, above 3 m/s, which is the galloping critical velocity [7], galloping instability takes place, and large amplitudes of OC voltage are generated by the harvester. The dispersion of experimental data increases markedly in this velocity range. When the harvester is excited by a wide band random base acceleration (band [10 60] Hz), before the critical galloping velocity, the amplitude of the rms of OC voltage increases; this is because the random excitation includes harmonic components at the natural frequency of the harvester. Above the critical galloping velocity, random base excitation does not cause an evident increase in OC voltage, taking into account the fact that, in this range of velocities, the standard deviation of data is large. Finally, when the band of random base excitation is narrower (band [10 30] Hz) and centered about the natural frequency of the harvester (19.3 Hz), there is a significant increase in the voltage generated before the galloping critical velocity. This phenomenon occurs because the rms value of base acceleration is constant (1 m/s^2^) and, when the band of excitation becomes narrower, the harmonic components at (or close to) the natural frequency of the harvester become more relevant (see Figure 5). Moreover, in this case, above the critical galloping velocity, random base excitation does not significantly increase the generated OC voltage.

### 4.2. Experimental Validation of the VIV

**Figure 7:**
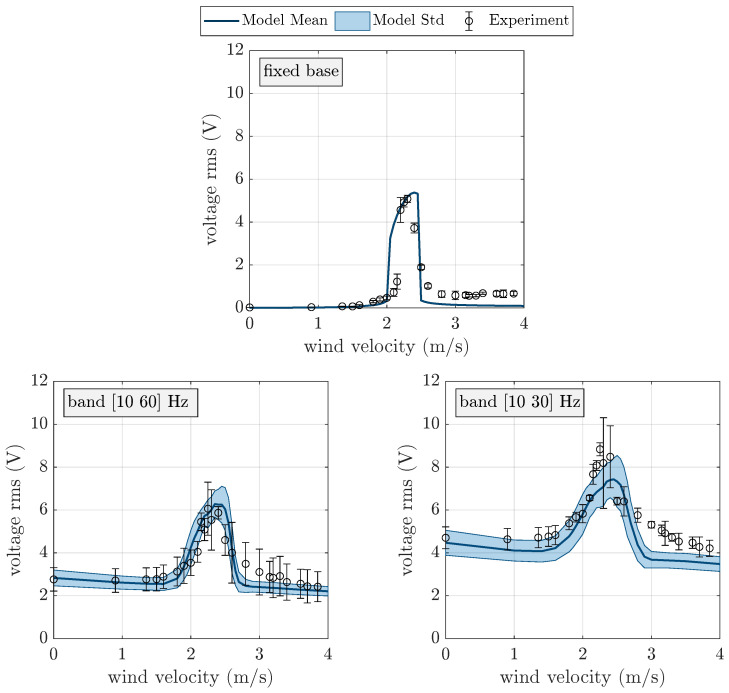
Experimental and numerical OC voltage of the VIV harvester without base excitation and with band-limited random base excitation (rms: 1 m/s^2^).

The results of the experimental tests carried out using the VIV harvester are depicted in Figure 7 in terms of open circuit (OC) voltage rms versus wind velocity. Three different working conditions are considered: only wind excitation; wind excitation plus random base excitation in the band [10 60] Hz and an rms value of 1 m/s^2^; wind excitation plus random base excitation in the band [10 30] Hz and an rms value of 1 m/s^2^. Since every experimental test was repeated three times, for every value of wind velocity, the mean value and the standard deviation of the rms voltage are represented.

The experimental results clearly show the lock-in peak. When the harvester base is steady, the rms value of OC voltage is almost zero before the lock-in condition and rather small after the lock-in condition. The dispersion of experimental data reaches large values at lock-in, since small perturbations (e.g., turbulence) are enough to alter the triggering of the lock-in phenomenon. In the presence of wide-band random base excitation (band [10 60] Hz), there are relevant rms values of OC voltage both before and after lock-in; this is because the random base excitation includes harmonic components at (or close to) the natural frequency of the harvester. Owing to the random base excitation, the dispersion of experimental data increases. If the band of random excitation is narrower (band [10 30] Hz) and centered about the natural frequency (19.3 Hz), the rms amplitude of OC voltage increases because more energy is concentrated in the band that includes the natural frequency of the harvester, with the rms value of base acceleration being constant (1 m/s^2^). The increase in amplitude due to VIV excitation at lock-in decreases with respect to the previous cases. This result is in agreement with the results presented in [28]. It is worth noting that before the lock-in phenomenon, when base excitation is dominant, the rms voltage generated by the VIV harvester is equal to that generated by the galloping harvester before the galloping critical velocity.

## 5. Numerical Simulations of Galloping

Equations (14) and (23) were transformed into a set of first-order differential equations and solved by means of the routine ODE45 of MATLAB (release 2024a).

When random base excitation is simulated, numerical calculations carried out in the same condition give different results, owing to the random nature of excitation. For this reason, 30 simulations were run for each wind velocity, and the rms and standard deviation were calculated. On the other hand, only one simulation is required for simulations with harmonic base excitation at a given frequency and amplitude.

A comparison of the numerical and experimental results obtained with the galloping harvester is shown in Figure 6. The numerical model is able to represent the most important features of harvester behavior, such as the critical galloping velocity and the large amplitudes at high wind velocity, when self-sustained galloping vibrations occur. The effect of random base excitation predicted by the numerical model is in agreement with the experimental results as well. The largest discrepancies between the numerical and experimental results occur near the critical galloping velocity; this is because, in the presence of base excitation, the numerical model predicts a smoother transition from the region of damped vibrations (for velocities smaller than the critical galloping velocity) and the region of self-sustained vibrations. The numerical model parameters used in the simulations are summarized in Table A1. It is worth noting that harvester damping $\zeta_{1}$ and the piezoelectric modal coupling term $\varphi_{1}$ were obtained from experimental tests [39], whereas the coefficients of the galloping force were found using the Den Hartog criterion [19] and the scientific literature.

**Figure 8:**
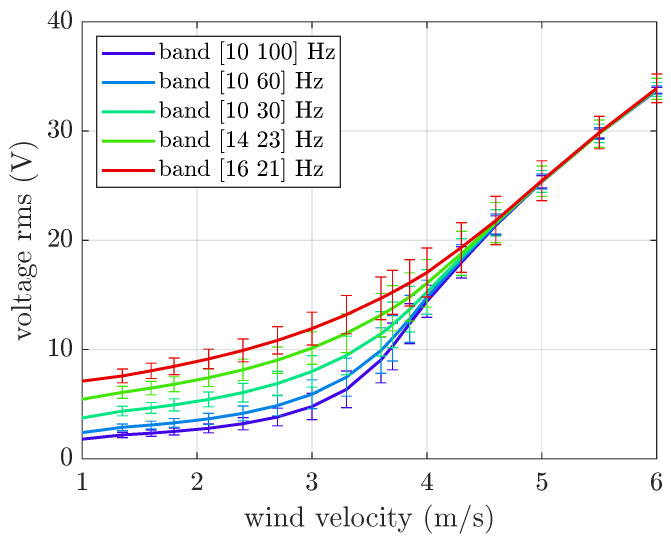
Mean value and standard deviation of the OC voltage generated by the galloping harvester. Numerical results with base excitation limited to various frequency bands and an acceleration rms equal to 1 m/s^2^.

Next, the numerical model was used to extend the range of investigation. Figure 8 shows the rms of OC voltage versus wind velocity for a larger range of wind velocities (up to 6 m/s), considering base excitations with frequency bands wider and narrower than the ones experimentally tested.

When wind velocity is below the critical galloping velocity, random base acceleration increases the generated voltage, and with a constant acceleration rms, the narrower the excitation band, the larger the increase in voltage. Conversely, above the critical galloping velocity, the contribution of self-sustained galloping vibrations dominates the response, and all the curves tend to the same value. The data variance reaches a maximum near the galloping critical velocity, when a transition occurs between damped and self-sustained vibrations.

In order to highlight the differences and similarities between harmonic and narrow-band excitation, a set of simulations with a harmonic base excitation below, at, and above the harvester’s natural frequency (i.e., 16 Hz, 19.3 Hz, and 21 Hz, respectively) was carried out. The rms of the acceleration was set to 1 m/s^2^.

**Figure 9:**
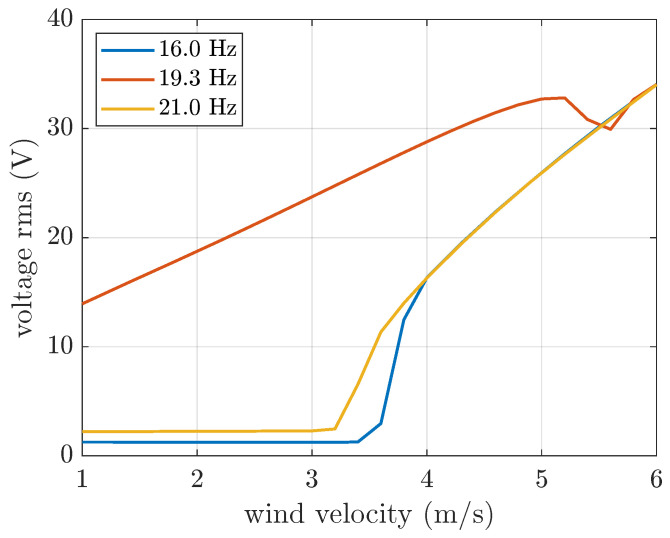
OC voltage generated by the galloping harvester subjected to harmonic base excitation at various frequencies (acceleration rms equal to 1 m/s^2^).

Figure 9 shows the effect of harmonic base excitation on the galloping harvester. When the harvester base is harmonically excited far from its natural frequency, the generated voltage is almost constant below the critical wind velocity. Above the critical wind velocity, galloping dominates, and the curves tend to the same value. When the base is harmonically excited at the harvester’s natural frequency, the voltage is higher at low wind velocity and shows a different trend. Indeed, the voltage increases almost linearly at low wind velocity and approaches the other curves at high wind velocity owing to the dominance of galloping.

Moreover, the curve at resonance shows a valley at about 5.5 m/s, which is associated with a quenching phenomenon ([19,40]). Indeed, the frequency of the base excitation is constant (19.3 Hz), whereas the natural frequency of the harvester subjected to galloping changes with wind velocity owing to the linear and non-linear terms in Equation (23), depending on cantilever deflection and wind velocity squared. The ratio between the frequency of base excitation and the natural frequency reaches the quenching value in a particular range of *U* [40], and the valley appears.

The comparison between Figure 8 and Figure 9 highlights that the voltage generated by band-limited random base excitation is a combination of the effects of various harmonic excitations, with a larger contribution due to harmonics close to resonance. The relevance of harmonics near resonance is more evident below the critical wind velocity due to the continuously increasing generated voltage.

**Figure 10:**
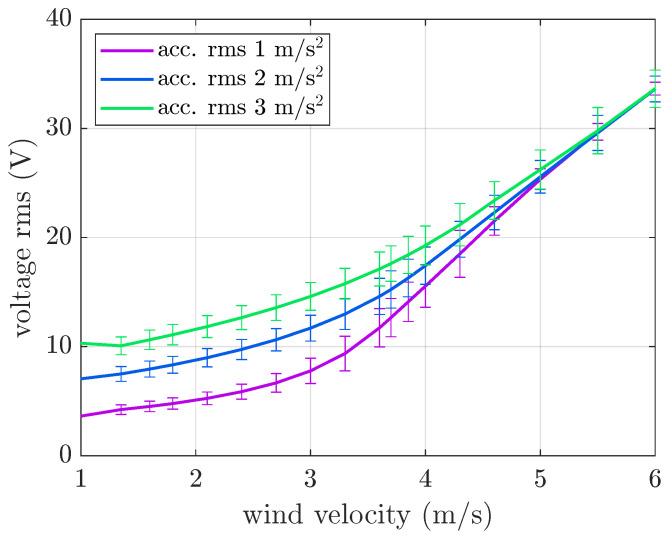
Mean value and standard deviation of the OC voltage generated by the galloping harvester. Numerical results with base excitation limited to the [10 30] Hz band and various acceleration rms values.

The effect of random base excitation with a fixed frequency band [10 30] Hz and amplitudes larger than those experimentally tested was also investigated. The numerical results, depicted in Figure 10, show that increasing the rms value of base acceleration generally increases the rms OC voltage; however, at high wind velocities, the effect of base acceleration amplitude tends to decrease, and all curves converge to the same values. A comparison between Figure 8 and Figure 10 shows that decreasing the bandwidth of random excitation (centered around resonance) with a fixed rms value and increasing the rms value of random excitation with a fixed bandwidth have similar effects.

## 6. Numerical Simulations of VIV

To simulate VIV excitation of the harvester equipped with a cylindrical bluff body, Equations (14) and (28) were transformed into a set of first-order differential equations and solved by means of the routine ODE45 of MATLAB (release 2024a).

**Figure 11:**
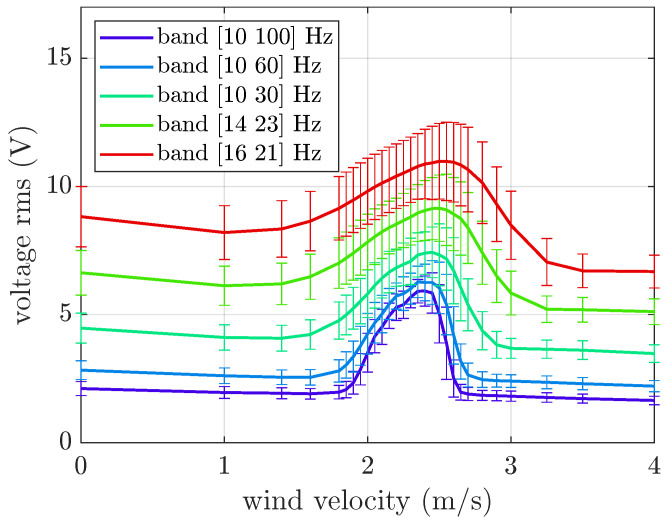
Mean value and standard deviation of the OC voltage generated by the VIV harvester. Numerical results with base excitation limited to various frequency bands and an acceleration rms equal to 1 m/s^2^.

Figure 7 shows the comparison between the numerical and experimental results obtained for the VIV harvester. The parameters of the numerical model adopted in the simulations are summarized in Table A2. When the harvester base is steady, the numerical model can capture the harvester’s dynamics and the lock-in phenomenon. The largest differences between the numerical and experimental data appear after lock-in. In the presence of random base excitation, the numerical model still fits the experimental data, both for wide-band and narrow-band excitation. For this reason, the numerical model was employed to extend the investigation to different bands of random base excitation. Figure 11 shows the numerical rms voltage against wind velocity.

When the band of random base excitation becomes narrower, there is a general increase in the generated voltage, and the lock-in phenomenon is always present. However, the lock-in phenomenon becomes less predominant. Indeed, for the [16 21] Hz band, the generated voltage rms at the lock-in is about 25% higher than the voltage generated by the base motion, whereas for the [10 100] Hz band, this percentage is about 180%. This means that there is a non-linear superposition of the two sources of excitation.

Moreover, the lock-in region becomes wider when the band of random base excitation is narrower because there are many large amplitude harmonics of base acceleration close to the natural frequency that are able to generate a relevant excitation of the harvester. Due to the presence of fluid-added damping (*c_f_*), the curves before and after lock-in decrease with increasing wind velocity (*U*); this effect is more evident as the band becomes narrower. A decrease in the OC voltage related to aerodynamic effects was also observed in the case of harmonic base excitation in [24].

In order to make a comparison between narrow-band random excitation and harmonic excitation, a set of simulations with harmonic base excitation below, at, and above the harvester’s natural frequency (i.e., 16 Hz, 19.3 Hz, and 21 Hz, respectively) was carried out. The rms of acceleration was set equal to 1 m/s^2^.

**Figure 12:**
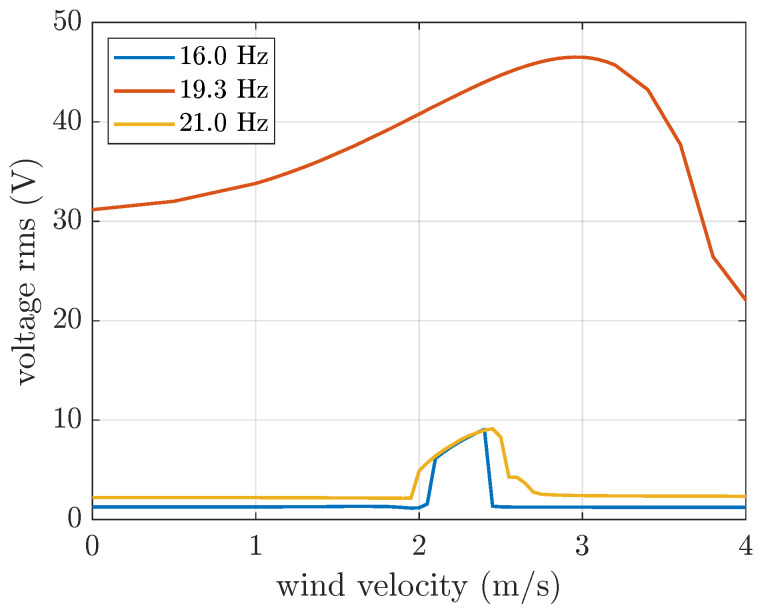
OC voltage generated by the VIV harvester subjected to harmonic base excitation at various frequencies (acceleration rms equal to 1 m/s^2^).

Figure 12 depicts the OC voltage generated by the VIV harvester under harmonic base excitation. Comparing Figure 11 and Figure 12, it can be observed that the voltage generated by band-limited random base excitation is a combination of the effects of various harmonic excitations. Harmonics near resonance make the largest contribution, increasing voltage generation outside lock-in and widening the lock-in peak. It is worth noting that the behavior of VIV harvesters subjected to harmonic base excitation close to their natural frequencies is highly sensitive to the excitation frequency, as shown in [28].

**Figure 13:**
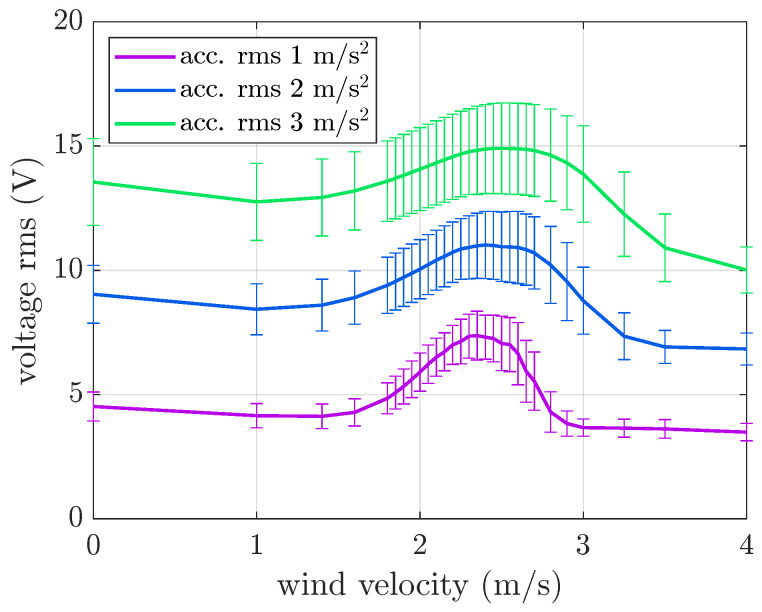
Mean value and standard deviation of the OC voltage generated by the VIV harvester. Numerical results with base excitation limited to the [10 30] Hz band and various acceleration rms values.

The model was also used to extend the VIV simulations to different rms levels of the random base excitation, with a fixed frequency band of [10 30] Hz. The results of the simulations with an acceleration rms of 1 m/s^2^, 2 m/s^2^, and 3 m/s^2^ are depicted in Figure 13 in terms of the rms OC voltage against wind velocity.

The results show that the voltage rms increases with the increase in the acceleration rms, and the lock-in condition is always present. However, as the acceleration level increases, the lock-in phenomenon becomes less pronounced. In fact, when the rms of the acceleration level is 3 m/s^2^, the generated voltage rms at lock-in is about 10% higher than that generated only by the base motion, whereas with an acceleration level of 1 m/s^2^, this percentage is about 63%. Therefore, there is a non-linear interaction between base excitation and VIV excitation, and large-amplitude random base excitation tends to cover the effect of wind excitation. These results are in agreement with those presented in [28]. Moreover, a reduction in the effect of VIVs at large base acceleration was also observed with harmonic base motion [24]. It is worth noting that increasing the acceleration rms with a fixed band and narrowing the band with a fixed acceleration rms have a similar effect on the system. Indeed, the effect of the base is dominated by the harmonics near the resonance, and the amplitudes of these harmonics increase in both cases.

Finally, both galloping and VIV harvesters, if properly tuned, are suited to collect low-velocity wind energy and represent an interesting alternative to small wind nanogenerators [41,42].

## 7. Conclusions

The galloping harvester generated large voltages due to aerodynamic excitation when the wind velocity was high and larger than the critical galloping velocity. In contrast, the VIV harvester generated large voltages due to aerodynamic excitation when the wind velocity was equal to or close to the lock-in velocity.

Numerical simulations corroborated by experimental tests show that random base excitation increases the voltage generated by both wind harvesters when the aerodynamic excitation is small. Conversely, the two devices exhibit different behavior in the range of wind velocities where aerodynamic excitation is important.

At high wind velocities, the performance of the galloping harvester is not affected by the amplitude and bandwidth of random base excitation; this means that galloping excitation far dominates the dynamics of the harvester. Near the lock-in velocity, the effect of VIV excitation and random base excitation adds up; however, there is an interaction between the two phenomena, and the contribution of VIV excitation tends to decrease if the amplitude of base excitation increases and if random excitation is concentrated in a narrow band centered about the resonance.

## Data Availability

The original contributions presented in this study are included in the article. Further inquiries can be directed to the corresponding author.

## Appendix A. Main Parameters of the Models

**Table A1:** Main parameters of the Galloping model.

Parameter	Value	Units
$m_{1}$	0.010	kg
$c_{1}$	0.025	Ns/m
$k_{1}$	151	N/m
$\varphi_{1}$	2.3·10^−4^	N/V
$C_{P}$	43	nF
*D*	18.6	mm
$L_{T}$	178	mm
ρ	1.225	kg/m^3^
$a_{1}$	4	−
$a_{3}$	−60	−

**Table A2:** Main parameters of the VIV model.

Parameter	Value	Units
$m_{1}$	0.010	kg
$c_{1}$	0.015	Ns/m
$k_{1}$	151	N/m
$\varphi_{1}$	2.3·10^−4^	N/V
$C_{P}$	43	nF
*D*	18.6	mm
$L_{T}$	178	mm
ρ	1.225	kg/m^3^
*A*	16	−
ϵ	0.5	−
$C_{V0}$	0.25	−
St	0.165	−
γ	0.7	−
